# Synergistic Effect of *Stauntonia hexaphylla* (Thunb.) Decne Fruit and Leaf on RAW 264.7 Osteoclast and MC3T3-E1 Osteoblast Differentiation

**DOI:** 10.3390/biom15060844

**Published:** 2025-06-10

**Authors:** Reshmi Akter, Muhammad Awais, Md Niaj Morshed, Jong Hak Kim, Byoung Man Kong, Dong Wook Lee, Sung Keun Choi, Chang Soon Lee, Jong Chan Ahn, Deok Chun Yang

**Affiliations:** 1 Graduate School of Biotechnology, College of Life Sciences, Kyung Hee University, Yongin-si 17104, Gyeonggi-do, Republic of Korea; reshmiakterbph57@gmail.com (R.A.); awaiskazmi@khu.ac.kr (M.A.); niajmorshed96@khu.ac.kr (M.N.M.); 2 Hanbangbio Inc., Yongin-si 16954, Gyeonggi-do, Republic of Korea; jhkim@rgk.co.kr (J.H.K.); dwlee5001@rgk.or.kr (D.W.L.); 3 Department of Oriental Medicinal Biotechnology, College of Life Sciences, Kyung Hee University, Yongin-si 17104, Gyeonggi-do, Republic of Korea; kong2167@naver.com; 4 Daedong Korea Ginseng Co., Ltd., 86, Gunbuk-ro, Gunbuk-myeon, Geumsan-gun 32718, Chungcheongnam-do, Republic of Korea; ddgc0815@ddkorea.co.kr (S.K.C.); hippo8270@ddkorea.co.kr (C.S.L.)

**Keywords:** *Stauntonia hexaphylla*, fruits, leaves, osteoporosis, osteoblast, osteoclast

## Abstract

*Stauntonia hexaphylla* (Thunb.) Decne (SH) is known for its anti-inflammatory, analgesic, antioxidant, and anti-osteoporotic properties. This study investigated the composition of SH leaves and fruits and assessed their individual and combined effects in an in vitro osteoporosis model. Extracts with varying leaf-to-fruit ratios (SH82, SH55, SH28) were evaluated in MC3T3-E1 and RAW264.7 cells to examine osteogenesis and bone resorption biomarkers. SH leaves were rich in chlorogenic acids (CGAs) and flavonoids, while fruits contained phenolic acids with minimal flavonoids. Leaf extracts exhibited superior antioxidant activity and ROS suppression. Both leaf and fruit extracts enhanced ALP activity, calcium deposition, and collagen formation in MC3T3-E1 cells, with leaf extracts demonstrating greater efficacy. Additionally, osteoblastogenesis-related genes were upregulated, while TRAP activity and osteoclast-related gene expression were significantly inhibited. The combined extract exerted a synergistic effect, with SH28 showing the most pronounced osteogenic enhancement and TRAP inhibition. Key components, including neo-CGA, CGA, rutin, and luteolin-7-O-glucoside, positively influenced ALP and TRAP activities. These findings highlight the potential of SH, particularly at a high leaf-to-fruit ratio, as a promising natural agent for osteoporosis prevention.

## 1. Introduction

The global aging population is rapidly expanding, with individuals aged 65 and older projected to reach 1.5 billion by 2050 [1]. This demographic shift has led to a rising prevalence of age-related diseases such as osteoporosis, which affects approximately 200 million people worldwide [2]. Osteoporosis is primarily caused by an imbalance between osteoblast-mediated bone formation and osteoclast-mediated bone resorption [3,4]. A key regulatory mechanism in bone remodeling is the OPG/RANKL/RANK signaling pathway. Osteoblasts express RANKL, which binds to RANK on osteoclast precursors, promoting osteoclast differentiation and activity [5,6]. Osteoprotegerin (OPG), a decoy receptor produced by osteoblasts and bone marrow stromal cells, inhibits this process by binding RANKL and preventing its interaction with RANK [7,8]. Dysregulation of this system, often associated with aging, contributes significantly to osteoporosis development [9]. While current treatments—including hormone replacement therapy, bisphosphonates, and calcitonin—are effective, their long-term use is limited due to adverse effects [10]. As a result, attention is shifting toward natural compounds with potential therapeutic benefits and lower toxicity profiles for osteoporosis management.

*Stauntonia hexaphylla* (Thunb.) Decne (SH), a member of the Lardizabalaceae family, is characterized by palmate leaves, tiny bell-shaped blooms, and edible fruits. It is widely distributed in China, Korea, and Japan [11]. Traditional oriental medicine has utilized various parts of SH, including its fruits, leaves, stems, and roots, for their analgesic, sedative, and diuretic properties [10].

Recent research has expanded our understanding of the bioactive compounds in SH, particularly its triterpene saponins, flavonoids, and phenolic acids. Notably, water-alcohol extracts from the leaves and fruits of SH have demonstrated anti-inflammatory activity through the inhibition of nitric oxide production and pro-inflammatory cytokines [12,13]. These studies identified triterpene glycosides from SH fruits and ethanolic extracts from leaves with promising pharmacological properties, suggesting that the plant’s anti-inflammatory effects are attributable to these phytochemicals. Furthermore, compounds such as oleanolic acid, found in SH and other medicinal plants, have been implicated in alleviating osteoporosis via the regulation of osteoblast and osteoclast function, supporting SH’s potential as an anti-osteoporotic agent [14].

Cheon et al. (2015) reported that a methanol extract of SH leaves promoted bone formation and inhibited bone resorption activity, possibly attributable to the flavonoids or phenolic acids present in the leaves [15]. Another study demonstrated that a combination of SH and *Vaccinium bracteatum* fruits alleviated menopausal symptoms, including hot flushes and postmenopausal osteoporosis [16]. These cumulative findings suggest that co-administration of substances with different effective ingredients may enhance efficacy in addressing osteoporosis [8,17]. Given the distinct composition of effective compounds in SH leaves and fruits, we hypothesize that their individual and combined effects on osteogenic activity may vary.

In the present study, we aimed to investigate the impact of SH leaf, fruit, and their combined mixture on osteoblastogenesis and osteoclastogenesis using an in vitro model. Additionally, we sought to elucidate the molecular mechanisms underlying these effects. This research can contribute to determining the optimal mixing ratio of SH fruit and leaves that maximizes efficacy in promoting bone health.

## 2. Materials and Methods

### 2.1. Sample Collection and Preparation

Fruits and leaves of SH were collected from a GAP-managed cultivation farm located in Jangheung-gun, Jeollanam-do, Republic of Korea. The fruit was a fully ripened fruit harvested in 2022, from late November to early December, and was cut into 1~2 cm thick pieces and dried at 45~50 °C for 24 h before use. The leaves were harvested at the same time as the fruit and included green young stems that grew that year. They were cut into 5~10 cm pieces and dried at 45~50 °C for 4~5 h using a far-infrared dryer.

The dried fruit was extracted twice for 6 h at 90 ± 3 °C with 10 times the volume of purified water, and the extract was filtered and concentrated to 30 brix before being used in the experiment. The dried leaves were extracted twice with 20 times the volume of purified water under the same conditions, filtered, concentrated with 30 Brix, and used as a sample. The mixed fruit and leaf samples were each extracted twice with 15 times the volume of purified water at the same temperature, filtered, concentrated to 30 brix, and used in the experiment. The fruit used in the mixed sample was steamed once, as in our previous report, to increase osteogenic activity.

### 2.2. Determination of Polyphenol Content

In the Total Phenolic Content (TPC) analysis, 30 μL of the extraction solution was combined with 150 μL of a 10% solution of 2N Folin-Ciocalteu reagent. Following vigorous shaking, the mixture was left at room temperature for 5 min. Subsequently, 160 μL of a 7.5% (*w*/*v*) sodium carbonate solution was introduced. The amalgamation was allowed to rest for 30 min in darkness, and the absorbance was then measured at 715 nm using a spectrophotometer. Utilizing a calibration curve with gallic acid as the reference standard facilitated the computation of TPC, expressed as μg, expressed as gallic acid equivalents (GAE)/mg of the extract.

### 2.3. Measurement of Free Radical Scavenging Ability

Antioxidant activity assessment was carried out using the DPPH (2,2-diphenyl-1-picrylhydrazyl) method [18]. In this assay, each sample underwent appropriate dilution, and 180 μL of a 0.2 mM DPPH solution was combined with 20 μL of the sample in a 96-well plate. Following a 30 min incubation period in the absence of light, absorbance was measured at 520 nm using an ELISA reader (Synergy-2, Bio-Tek Instruments, Inc., Vinooski, VT, USA). The antioxidant capacity was determined and expressed as μgGAE/g dry weight, representing the radical scavenging ability.

### 2.4. HPLC Analysis

The quantification and/or identification of phytochemicals in fruits and leaves of SH was conducted using the following methods. Namely, an HPLC (High-Performance Liquid Chromatography) system (Agilent Infinity 1260, Agilent Technologies, Santa Clara, CA, USA) was employed. The system comprised an Agilent 1260 Infinity Quaternary Pump (G1311B), Agilent 1260 Infinity Standard Auto Sampler (G1329B), Agilent 1260 Infinity Column Thermostat Compartment (G1316A), and Agilent 1260 Infinity Variable Wavelength Detector (G1314F), and a ZORBAX Eclipse Plus C18 (Agilent Technologies, Santa Clara, CA, USA, 4.6 mm × 10 mm, 5 μm) served as the stationary phase. HPLC conditions included an injection volume of 5 μL, a temperature of 35 °C, a flow rate of 1 mL/min, and a wavelength of 260 nm. The mobile phase consisted of solvent A (0.4% phosphoric acid) and solvent B (acetonitrile), with an initial elution composition of 95% A and 5% B. Gradient elution was performed as follows: (0~10 min, 5~9% B; 10~30 min, 9~9% B; 30~60 min, 9~30% B; 60~62 min, 30~50% B).

Authentic standards for CGA, neo-CGA, crypto-CGA, quercetin, apigenin, rutin, naringin, hesperidin, luteolin-7-O-glucoside, prunin, eriodictyol, gallic acid (sigma), ellagic acid, caffeic acid, ferulic acid, tannic acid, catechin (sigma), and epicatechin (sigma) were purchased from Sigma-Aldrich (St. Louis, MO, USA) and 5-HMF were sourced from Wuhan ChemFaces Biochemical Co., Ltd. (Wuhan, China).

### 2.5. Cell Viability Assay

MC3T3-E1 pre-osteoblast and RAW264.7 macrophage cell lines were seeded at a density of 1 × 10^4^ cells per well in 96-well culture plates. After overnight incubation, the cells were exposed to different concentrations of SH extracts (31.25, 62.5, 125, 250, and 500 µg/mL) for durations spanning 24 to 48 h. Cell viability was assessed using the MTT (3-(4,5-dimethylthiazol-2-yl)-2,5-diphenyltetrazolium bromide) assay after a 2-day incubation period.

For the MTT assay, 20 μL of MTT solution (5 mg/mL) was added to each well, and the plates were incubated for 3 to 4 h. After incubation, MTT solutions were removed, and 100 µL of dimethyl sulfoxide (DMSO) was added to dissolve the formazan product. Subsequently, absorbance was measured at 570 nm using a microplate reader (BioTek Instruments, Inc., Winooski, VT, USA). Relative cell viability was expressed as the percentage relative to untreated control cells.

### 2.6. Osteoblast Cell Culture and Differentiation

The MC3T3-E1 pre-osteoblast cell line (RCB1126, derived from C57BL/6 mouse calvaria) was obtained from the RIKEN Cell Bank in Tsukuba, Japan. Cells were cultured in α-MEM medium (Gibco-BRL, Grand Island, NY, USA) supplemented with 10% heat-inactivated fetal bovine serum (Gibco) and 1% penicillin-streptomycin (Gibco), constituting the complete medium (CM). Cultivation took place at 37 °C in a humidified 5% CO_2_ incubator provided by Thermo Electron Corporation in the USA.

Upon reaching 80% to 90% confluence, cells were seeded in a 12-well plate at a density of 5 × 10^3^. To induce cell differentiation, 10 mM glycerophosphate and 50 µg/mL ascorbic acid were added to the CM, creating the differentiation medium (DM). This induction was maintained for an additional 6 to 24 days to support and enhance cellular differentiation.

### 2.7. Alkaline Phosphatase (ALP) Activity Assay

The ALP activity of differentiated osteoblasts was evaluated using an ALP assay kit obtained from Sigma Chemical in St. Louis, MO, USA, following the provided manufacturer’s instructions. Briefly, MC3T3-E1 cells underwent differentiation in a 12-well plate using 10 mM glycerophosphate and 50 µg/mL ascorbic acid in complete medium (CM), with or without SH (25, 50, 100 μg/mL). Following a 12-day differentiation period, cell monolayers were lysed with 0.1% Triton X-100/PBS, followed by three PBS washes. Subsequently, cell lysates were centrifuged at 12,000 rpm for 5 min at 4 °C, and the supernatant was collected in 1.5 mL tubes for ALP activity measurement.

The cell supernatant and pNPP substrate were combined in a 96-well plate and incubated for 10 min at 37 °C. The enzymatic reaction was stopped using the provided stop solution after the specified incubation time. Finally, optical density (OD) values at 405 nm were measured using an ELISA reader (Bio-Tek Instruments, Inc., Winooski, VT, USA). Additionally, the ALP activity was normalized using a BCA kit from Sigma Chemical in St. Louis, MO, USA.

### 2.8. Alizarin Red Staining

MC3T3-E1 cells underwent differentiation in the presence or absence of SH extracts (25, 50, 100 μg/mL) following established protocols. After a 24-day differentiation period, the cells were thoroughly rinsed twice with PBS. Subsequently, they were fixed with 4% paraformaldehyde for 20 min and stained with a 2% Alizarin Red S [19] dye solution from Sigma-Aldrich, adjusted to pH 4.2, at room temperature for 10 min. The mineralization nodules in the extracellular matrix were captured using a digital camera attached to an inverted microscope (Nikon Instruments, Melville, NJ, USA).

For ARS staining analysis, a resolving solution consisting of 10% acetic acid and 20% methanol was utilized to dissolve the cells. After drying, the resulting liquid was transferred to a 96-well plate 15 min later. The absorbance was then measured at a wavelength of 450 nm using an ELISA reader (Epoch TM Microplate Spectrophotometer: BioTek Instruments Inc., Winooski, VT, USA). Each reaction was performed in triplicate.

### 2.9. Collagen Content

MC3T3-E1 cells were cultured using the same procedures employed for the ALP assays to evaluate cellular collagen levels. After an overnight incubation and reaching confluence, cells were exposed to differentiation medium (DM) with or without SH extracts (25, 50, 100 μg/mL) for an additional 12 days. The culture medium was refreshed every two days during this period.

Following the 12-day treatment, the collagen content was quantified using a Sirius-Red-based colorimetric assay. In a concise process, cells were rinsed twice with PBS and then exposed to Bouin’s solution for 1 h. Subsequently, the Bouin’s solution was removed, and the cells were washed with running tap water for ten to fifteen minutes. The plates were left to air-dry before staining with a reagent containing Sirius Red dye for an additional 1 h with gentle agitation. Following this staining period, 0.01 N HCl was used to wash the cells and remove any residual dye. After dissolving the dyed material in 0.1 N NaOH, the absorbance of the resulting solutions was measured with a microplate reader at 550 nm.

### 2.10. Osteoclast Cell Culture and Differentiation

RAW264.7 mouse macrophage-like cells were obtained from Ginseng Bank at Kyung Hee University, Korea. These cells were cultured in a complete medium (CM) consisting of 89.5% Dulbecco’s modified Eagle’s medium, 10% fetal bovine serum (FBS), and 100 U/mL penicillin-streptomycin. The culture was maintained in a CO_2_ incubator at 37 °C. Cells were seeded in 12-well plates at a density of 5 × 10^3^ cells/well, with or without the addition of 50 ng/mL of RANKL when they reached 80% to 90% confluence. Subsequently, the cells were cultured for an additional 3–7 days, and multinucleated osteoblast cells became visible by day 7.

### 2.11. TRAP Activity Assay

RAW264.7 cells were cultured and prompted to differentiate with 100 ng/mL RANKL, either in the presence or absence of SH extract (25, 50, 100 μg/mL), over a 5 to 7-day period. After osteoclast cell differentiation, the cell monolayers were washed with PBS and subsequently centrifuged at 12,000 rpm for 5 min. The cells were then lysed with 0.5% Triton X-100, and the resulting supernatant was employed for activity quantification using a TRAP staining kit from Sigma Chemical in St. Louis, MO, USA, following the manufacturer’s protocol provided. Cell counting was used to examine TRAP-positive cells under a light microscope in at least five random fields.

### 2.12. Measurement of Reactive Oxygen Species (ROS)

Reactive oxygen species (ROS) production was assessed using ROS detection reagents (Invitrogen, Carlsbad, CA, USA). Briefly, 2 × 10^4^ cells per well were seeded in a 96-well plate and treated with 50 ng/mL recombinant mouse RANKL in combination with varying concentrations of resveratrol (SH; 100 μg/mL). On day 5, cells were washed with PBS and incubated with 10 μM carboxy-H_2_DCF-DA at 37 °C for 1 h. Fluorescence intensity, indicating intracellular ROS levels, was measured at 0, 10, and 30 min using a fluorescence multi-well plate reader (excitation: 492 nm, emission: 520 nm).

### 2.13. RNA Isolation and Real-Time Reverse Transcription-PCR (qRT-PCR) Analysis

Following the differentiation and treatment of MC3T3-E1 and RAW264.7 cells with or without SH F extracts (100 μg/mL), total RNA extraction was carried out using TriZol LS reagents (Invitrogen, Carlsbad, CA, USA) as per the manufacturer’s instructions. Additionally, 20 μL of cDNA was synthesized from 2.5 ng of RNA utilizing a RevertAid First Strand cDNA Synthesis Kit from Thermo Fisher Scientific, Waltham, MA, USA. The synthesis process involved incubating the mixture at 42 °C for 45 min, followed by a 70 °C incubation for 5 min, following the manufacturer’s guidelines. The entire procedure was conducted within a PCR-clean environment.

For the assessment of gene expression, real-time reverse transcription-PCR (qRT-PCR) was employed using an Invitrogen SYBR Green qPCR Super Mix UDG kit and an R-Corbett Rotor-Gene Model 6000 (Mortlake, NSW 2137, Australia). The relative expression of gene-specific products was evaluated and normalized to the corresponding β-actin levels, utilizing the 2^−∆∆Ct^ method. These results were validated through three independent experiments. Detailed primer information can be found in Supplementary Table S1.

## 3. Results

### 3.1. Comparison of Ingredients of SH Fruits and Leaves

To compare the bioactive compounds between the SH leaves (L) and SH fruits (F), a qualitative comparison was performed using HPLC (Supplementary Figures S1 and S2). The components contained in the leaves and fruits of SH differed remarkably. The HPLC pattern of the absorption components in the ultraviolet region showed the differences between fruit and leaf components more clearly (Supplementary Figure S2). That is, the leaves mostly showed high peaks across a broad spectrum, while the fruits showed high levels of phenolic acids at early retention times. In the case of the leaves, several flavonoid compounds not present in the fruit, such as rutin, naringin, luteolin-7-o-glucoside (L-7-G), qurcetin-3-glucoside and prunin, were found.

The total phenolic compounds (TPC) were 1.02 ± 0.04 (mg GAE/g) in the fruit and 7.66 ± 0.07 (mg GAE/g) in the leaves, which was about 7 times higher in the leaves. On the other hand, the presence of 5-HMF, which was not detected in the leaves, was confirmed in the fruit. In particular, the CGA content including neo-CGA and crypto-CGA, which is used as an indicator of SH, was about 6 times higher, at 2395 ± 20 µg/g in the leaves compared to 360 ± 12 µg/g in fruit, as shown in Table 1.

### 3.2. Comparison of Antioxidant Activities of SH Fruits and Leaves

To compare the antioxidant activities between the SH L and F, DPPH and ROS generation was performed. The free radical scavenging ability of the fruits and leaves by DPPH assay was 20.41 ± 0.02 mg RE/g and 26.26 ± 0.02 mg RE/g, respectively, with the leaves also significantly higher than the fruits (Supplementary Figure S3A).

Supplementary Figure S3B compares the ability of the SH fruit and leaf extracts to inhibit intracellular reactive oxygen species production in Raw 264.7 cells. The fruit extract inhibited ROS production by 53%. However, the leaf extract inhibited this by up to 72%, which was much higher than that of the fruit.

### 3.3. Comparison of the Effect on ALP Activities of SH Fruits and Leaves

ALP is an enzyme secreted by osteoblasts, serving as a direct indicator of osteoblast differentiation. Initially, a cell proliferation assay was conducted to determine suitable concentrations of SH F and L extracts for the viability of MC3T3-E1 cells (Supplementary Figure S4A–C).

After a 24 h exposure to extracts SH L and SH F, there was a dose-dependent increase in cell proliferation (Supplementary Figure S4A). However, this proliferative effect did not persist after a 48 h treatment (Supplementary Figure S4B). Notably, at higher concentrations, the proliferative effect of the steamed SH extracts was diminished compared to the 24 h treatment. Consequently, concentrations up to 100 µg/mL of SH extracts were chosen for the subsequent experiments.

Photomicrographs taken during the observation period revealed that MC3T3-E1 cells displayed a flat, polygonal morphology with uniformly thin, smooth extended cytoplasm. Additionally, under an inverted phase-contrast microscope, the cells exhibited a typical spindle-shaped morphology with a fibroblastic appearance (Supplementary Figure S4C).

**Figure 1:**
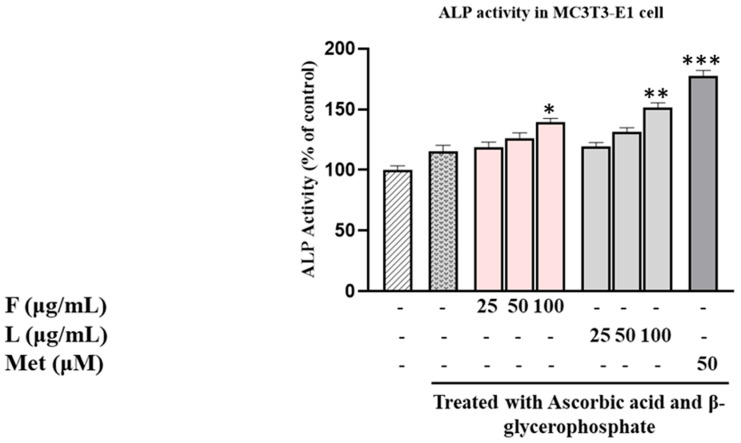
Evaluation of SH L and SH F impact on ALP (alkaline phosphatase) activity in MC3T3-E1 cells over a 7-day culture period with different concentrations (25 to 100 μg/mL). The presented data represent the mean ± standard deviation (SD) from three independent experiments. Statistical analysis indicated significant differences, marked as * *p* < 0.05, ** *p* < 0.01, *** *p* < 0.001 in comparison with the reference groups treated with ascorbic acid and β-glycerophosphate, as indicated.

Moving on to the assessment of ALP activity in MC3T3-E1 cells (Figure 1), the results indicated that the SH F extract increased ALP activity in a concentration-dependent manner. Specifically, the ALP activity rose by 18% at 25 μg/mL, 26% at 50 μg/mL, and 39% at 100 μg/mL compared to the control group only treated with dilution medium. In contrast, the SH L extract demonstrated a more robust effect, with ALP activity increasing by 19% at 25 µg/mL, 30% at 50 µg/mL, and 51% at 100 µg/mL, respectively. These findings underscore the distinct impact of SH F and L extracts on ALP activity in MC3T3-E1 cells, suggesting potential implications for osteoblast differentiation.

### 3.4. Comparison of the Effect on Mineralization and Collagen Content of SH Fruits and Leaves

**Figure 2:**
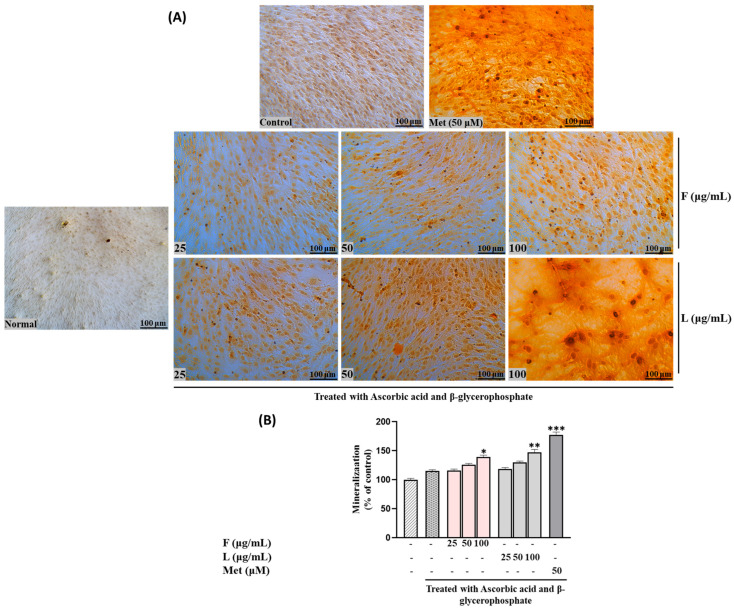
Effects of SH L and SH F and different ratios of extracts on mineralization in MC3T3-E1 cells. Cells were subjected to treatment with or without differentiation media containing extracts at concentrations ranging from 25 to 100 μg/mL for a duration of 12 days. (**A**) Alizarin red staining was conducted, and the results were visualized through microscopy at 100× magnification. (**B**) Mineralization (%) was quantified by measuring absorbance at 562 nm. The presented data represent the mean ± standard deviation from three independent experiments. Statistical analysis indicated significant differences, represented as * *p* < 0.05, ** *p* < 0.01, *** *p* < 0.001 in comparison with the respective ascorbic acid and β-glycerophosphate-treated group.

We investigated the impact of SH F and L extracts on intracellular mineral formation, specifically focusing on the accumulation of calcium, and collagen formation, serving as additional biomarkers to assess their osteotropic activity. As illustrated in Figure 2, calcium deposition, visualized through Alizarin Red S staining in MC3T3-E1 cells, exhibited a gradual dose-dependent increase in both treatment groups.

To elaborate, at lower concentrations (25 μg/mL) of the steamed SH F treatment, the calcium deposition was relatively low (116.231± 2.32418). However, as the concentration was increased to 50 and 100 μg/mL, this rose to 125.8946 ± 2.361123 and 139.4602 ± 3.130763, respectively. Notably, the leaf extract demonstrated a higher mineralization percentage than the fruit extract. At 25 μg/mL of SH L extract, the mineralization increased to 118.8468 ± 2.315338, followed by 129.9332 ± 2.39116 and 147.0308 ± 5.30361 at 50 and 100 μg/mL, respectively.

**Figure 3:**
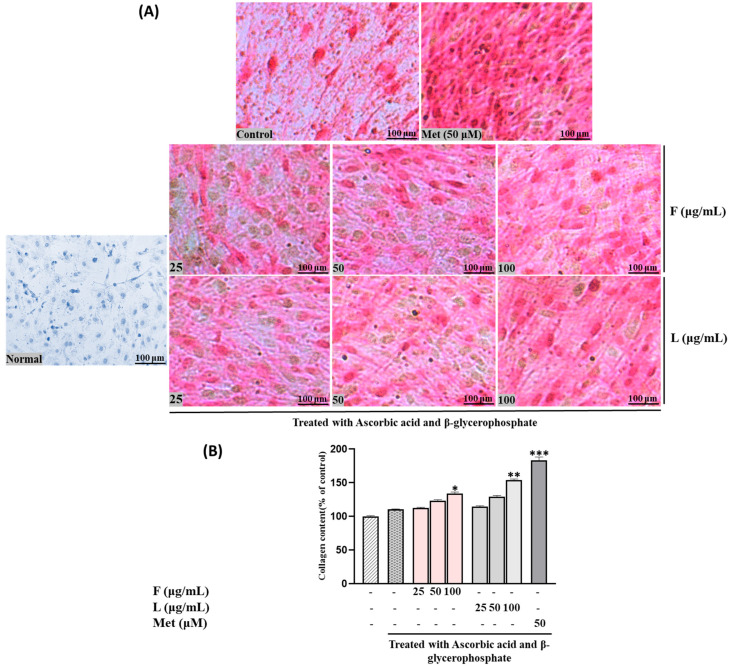
Effects of SH L and SH F extracts on collagen production in MC3T3-E1 cells. Cells were subjected to treatment with or without differentiation media containing extracts at concentrations ranging from 25 to 100 μg/mL for a duration of 12 days. (**A**) Picro-sirius red staining was carried out, and the outcomes were visualized through microscopy at 100× magnification. (**B**) Collagen content was determined by measuring absorbance at 550 nm. The presented data represent the mean ± standard deviation from three independent experiments. Statistical analysis indicated significant differences, represented as * *p* < 0.05, ** *p* < 0.01, *** *p* < 0.001 in comparison with the respective ascorbic acid and β-glycerophosphate-treated group.

Collagen production, another crucial biomarker for bone formation alongside mineralization, exhibited a similar pattern to calcium accumulation. In other words, both SH F and L extracts increased collagen production, demonstrating comparable activity to ALP levels (Figure 3). These findings suggest that both extracts may contribute to enhanced bone formation through the stimulation of mineralization and collagen production in MC3T3-E1 cells.

### 3.5. Comparison of the Effect on TRAP Activities of SH Fruits and Leaves

Next, we examined the impact of SH L and F on osteoclast cell survival and osteoclastogenesis using RAW264.7. Supplementary Figure S5A–C present the survival rates 24 and 48 h post-treatment with varying concentrations of leaf and fruit extracts. While the cell viability tended to decrease with higher concentrations, no toxicity was observed up to 500 µg/mL within the initial 24 h. After 48 h, the cell survival was approximately 87% up to 125 µg/mL. Consequently, the concentration of 100 µg/mL was selected for further investigation.

**Figure 4:**
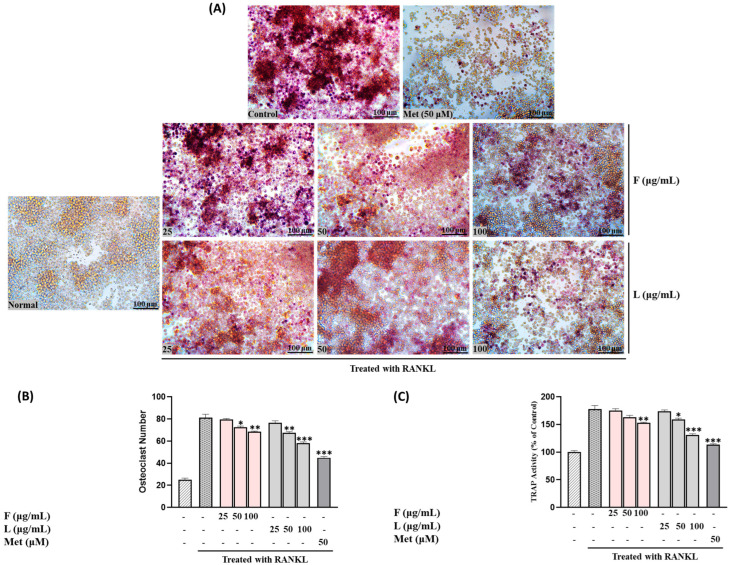
(**A**) Osteoclast differentiation induced by RANKL and influenced by SH L and SH F concentrations (25 to 100 μg/mL) over a 7-day period. (**B**) Osteoclast formation: Multinucleated osteoclast-like cells were observed at 100× magnification through light microphotography. Scale bars, 100 μm. (**C**) Enumeration of TRAP-positive multinucleated cells as indicative of osteoclasts. Statistical analysis indicated significant differences, represented as * *p* < 0.05, ** *p* < 0.01, *** *p* < 0.001 compared to the specified RANKL-treated group.

TRAP activity was assessed following treatment with fruit and leaf extracts at concentrations of 25, 50, and 100 µg/mL. Both extracts exhibited a concentration-dependent inhibition of TRAP activity, with the leaf extract consistently demonstrating significantly higher inhibitory effects compared to the fruit extract (*p* < 0.01 at 100 µg/mL) (Figure 4C).

Figure 4B,C illustrates the impact of SH F and L extract treatment on the number of osteoclasts and TRAP-positive cells in the RAW264.7 cell culture system. Treatment with both extracts led to a significant reduction in the quantity of osteoclasts and TRAP-positive cells. Moreover, the reduction was more pronounced with increasing treatment concentration, particularly in the leaf extract group.

### 3.6. Preparation of Fruit and Leaf Mixture

To assess the impact of a combined SH F and L mixture on bone metabolism, we examined alterations in the activity of biomarkers associated with both bone formation and resorption, utilizing extracts blended at specific ratios. Table 2 details the mixing ratios of fruit and leaves, along with the solid content and chlorogenic acid (CGA) content for each extract. Notably, the fruit extract exhibited a slightly higher solid content of 436 mg/g compared to the leaves at 341 mg/g, while the CGA content was significantly greater in the leaves, as previously observed. Furthermore, the solid and CGA contents were maintained based on the proportion of fruit and leaves in the mixture.

In order to understand qualitatively the mixed ratios of SH L and F, we carried out HPLC analysis of ratios (Supplementary Figure S6). Within the chromatograms, red-colored arrows highlight components abundant in fruits, green-colored arrows indicate constituents prevalent in leaves, and brown-colored ones denote representative ingredients present in both. The peak heights accurately reflect the mixing ratio of fruit and leaves. Specifically, ① represents 5-HMF, ② is neo-CGA, ③ corresponds to CGA, ④ signifies crypto-CGA, ⑤ indicates rutin, and ⑥ denotes luteolin-7-O-glucoside, all of which were confirmed through comigration with authentic standard materials.

### 3.7. Effect of Mixed Extracts of SH on Bone Formation and Bone Resorption

The impact of extracts derived from SH F and L, as well as samples mixed in ratios of 8:2, 5:5, and 2:8, on the viability of MC3T3-E1 cells and RAW264.7 cells was evaluated. For MC3T3-E1 cells, the growth remained unaffected even at a high concentration of 500 µg/mL when treated with fruit, leaf, or mixed sample extracts. However, at lower concentrations of 32.25 and 125 µg/mL of leaf and fruit extracts, a slight inhibition in cell growth was observed after 24 h, see Supplementary Figure S7A–C, which interestingly reversed upon 24 h of culture. In the case of RAW264.7 cells, the cell growth exhibited a slight inhibition with increasing treatment concentration for all three samples, reaching about 25% inhibition at 500 µg/mL, see Supplementary Figure S8A–C.

**Figure 5:**
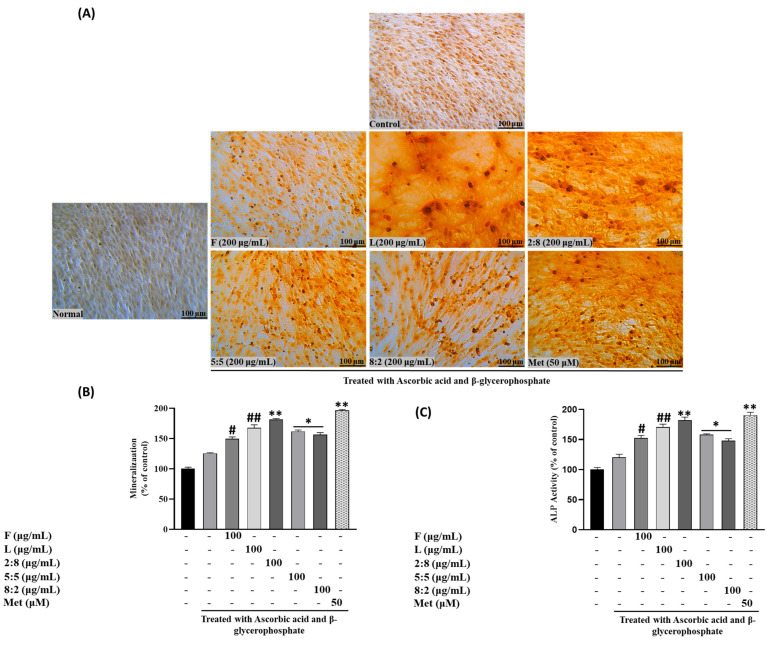
Effects of SH L and SH F mixtures on mineralization in MC3T3-E1 cells. Cells were subjected to treatment with or without differentiation media containing extracts at a concentration of 100 μg/mL for a duration of 12 days. (**A**) Alizarin red staining was conducted, and the results were visualized through microscopy at 100× magnification. (**B**) Mineralization (%) was quantified by measuring absorbance at 562 nm. (**C**) Evaluation of the impact of SH L and SH F mixtures on ALP (alkaline phosphatase) activity in MC3T3-E1 cells over a 7-day culture period. The presented data represent the mean ± standard deviation from three independent experiments. Statistical analysis indicated significant differences, represented as # *p* < 0.05, ## *p* < 0.01 in comparison with the respective ascorbic acid and β-glycerophosphate treated group and * *p* < 0.05, ** *p* < 0.01, in comparison with the respective leaf treated group.

The results indicated that the effects of SH L and F extracts, as well as mixed extracts, on ALP activity with the treatment of 100 µg/mL of F extract increased ALP activity by 39.5%, and the L extract at the same concentration led to a 50% increase compared to the control. Mixed extracts, such as SH82 and SH55, exhibited enhanced activity proportional to the higher concentration of leaves. Notably, SH28 demonstrated a significant increase of 70% (*p* < 0.001), surpassing the activity of the leaf extract alone (Figure 5A–C).

**Figure 6:**
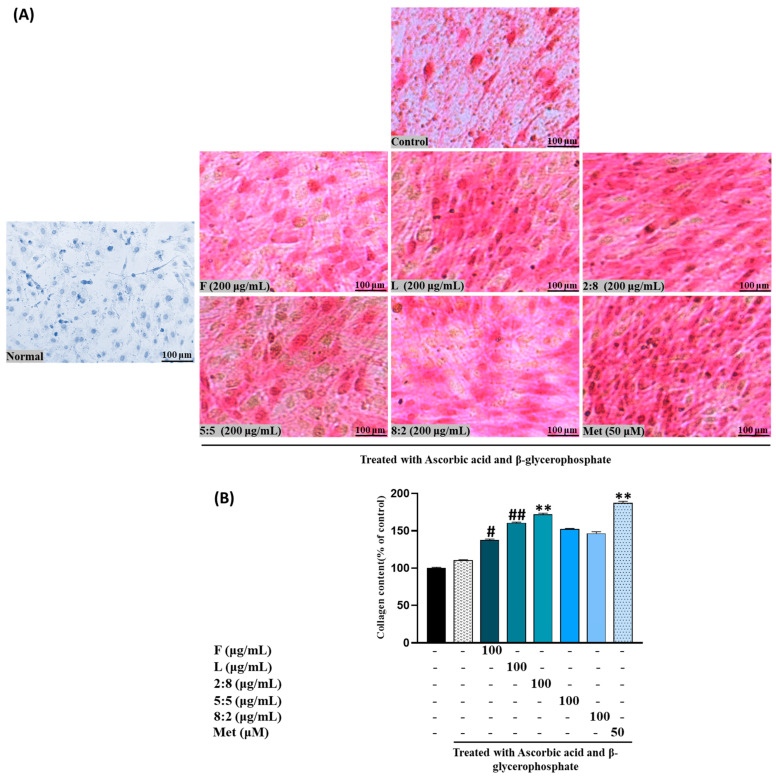
Effects of SH L and SH F mixtures on collagen production in MC3T3-E1 cells. Cells were subjected to treatment with or without differentiation media containing mixture for a duration of 12 days. (**A**) Picro-sirius red staining was carried out, and the outcomes were visualized through microscopy at 100× magnification. (**B**) Collagen content was determined by measuring absorbance at 550 nm. The presented data represent the mean ± standard deviation from three independent experiments. Statistical analysis indicated significant differences, represented as # *p* < 0.05, ## *p* < 0.01 in comparison with the respective ascorbic acid and β-glycerophosphate treated group and ** *p* < 0.01, in comparison with the respective leaf treated group.

Following ARS staining, the nodules of mineralized calcium deposition displayed a striking orange-red color. The synergistic impact of mixed extract SH28 on bone formation was further evident in calcium nodule production through ARS staining and collagen production tests via PSR staining, as depicted in Figure 6A,B.

**Figure 7:**
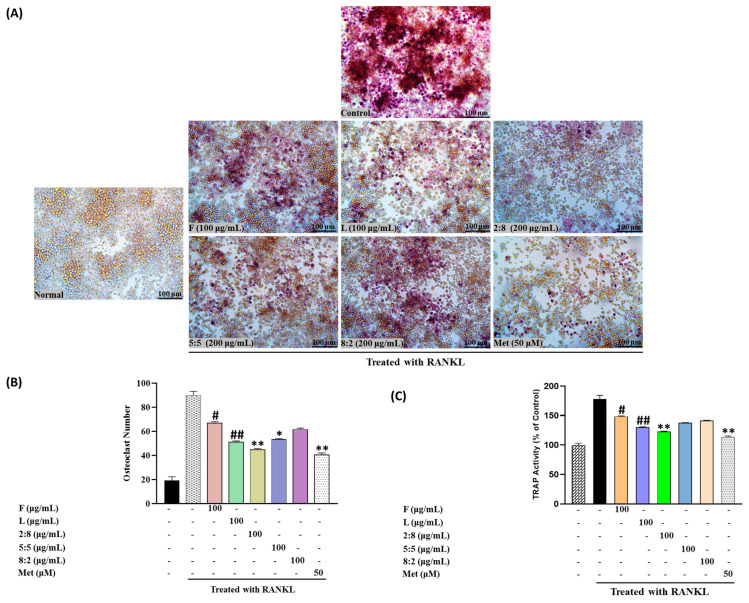
(**A**) Osteoclast differentiation induced by RANKL and influenced by SH L and SH F mixtures over a 7-day period. (**B**) Osteoclast formation: Multinucleated osteoclast-like cells were observed at 100× magnification through light microphotography. Scale bars, 100 μm. (**C**) Enumeration of TRAP-positive multinucleated cells as indicative of osteoclasts. Statistical analysis indicated significant differences, represented as # *p* < 0.05, ## *p* < 0.01 in comparison with the respective RANKL treated group and * *p* < 0.05, ** *p* < 0.01, in comparison with the respective leaf treated group.

Figure 7 depicts the results concerning the effect on TRAP activity measured in RAW264.7 cells. Exposure to F, L, and mixed extracts at a concentration of 100 μg/mL led to a decrease in TRAP activity and the number of TRAP-positive multinucleated cells. Notably, SH28 exhibited the highest inhibitory activity, surpassing both the control group and the leaf extract treatment group alone (*p* < 0.05).

### 3.8. Comparison of the Effect on Osteoblastogenesis and Osteoclastogenesis Related Genes Expression of SH Fruits and Leaves and Mixture

We investigated the influence of extracts from SH F and L, as well as their combinations, on the sequential expression and regulation of specific biomarkers associated with both bone formation and bone destruction. In MC3T3-E1 cells treated with ascorbate and ß-glycerophosphate, we scrutinized distinct bone formation biomarkers, including ALP, RANKL, Runx2, type I collagen (Coll-I), and osteoprotegrin (OPG). Furthermore, in RAW264.7 cells treated with RANKL, we investigated bone resorption-related factors such as TRAP, TRAF6, RANK, Ctsk, c-fos, and NFATc1. These proteins serve as pivotal markers for pre-osteoblast differentiation during bone formation, and osteoclast differentiation and proliferation during bone resorption.

**Figure 8:**
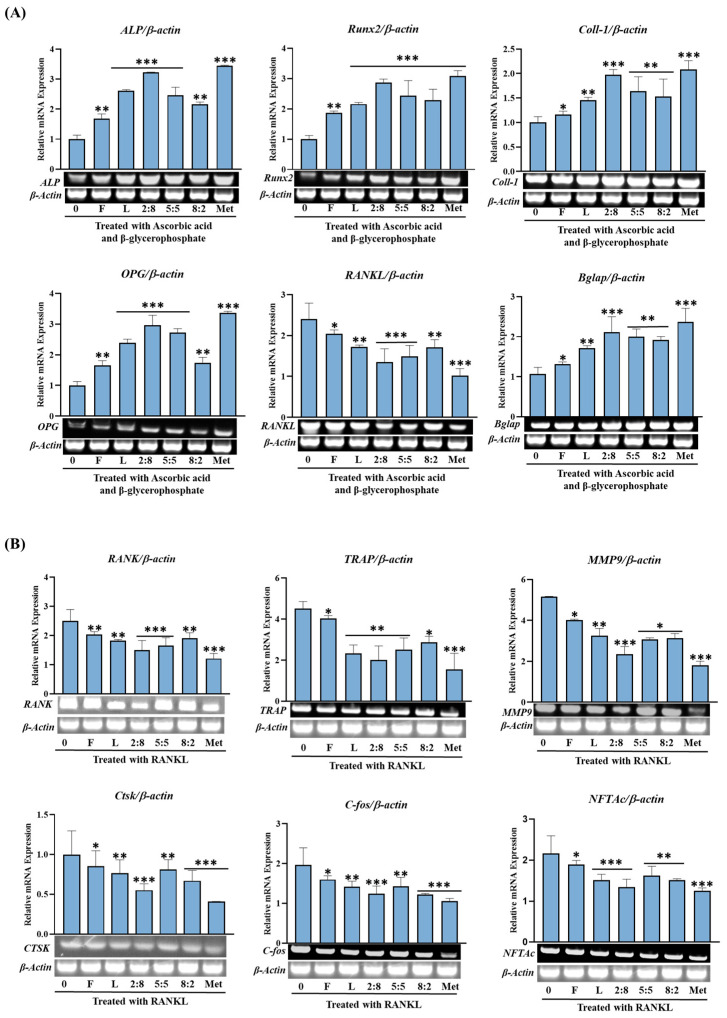
Effects of SH L and SH F mixtures on osteoblast differentiation in MC3T3-E1 cells. Cells were treated with or without differentiation media containing the extracts for a duration of 14 days. (**A**) Analysis of the relative mRNA expression levels of osteoblast markers (Alp, Runx2, Col-I, BGLAP, OPG, and RANKL) using real-time PCR. (**B**) Examination of the mRNA expression of transcriptional factors associated with osteoclast differentiation. RAW 264.7 cells were exposed to RANKL and SH F extracts for 7 days, and the expression of osteoclast differentiation marker genes (TRAP, RANK, TRAF6, MMP9, c-Fos, CtsK, NFTAc) was measured using real-time PCR. Statistical analysis revealed significant differences, denoted as * *p* < 0.05, ** *p* < 0.01, *** *p* < 0.001 compared to the corresponding ascorbic acid and β-glycerophosphate-treated group and RANKL-treated group, respectively.

In Figure 8, we present a comparison of the relative activities measured at a concentration of 100 µg/mL for each SH leaf and fruit extract, as well as their mixtures. Concerning osteogenesis-related gene expression, both samples demonstrated a robust promoting activity. The leaf extract consistently exhibited a more pronounced enhancement of the expression of these proteins compared to the fruit extract, except for RANKL, which showed downregulation, indicating an inhibitory effect on bone resorption. Notably, the expression of genes such as ALP, Runx2, and OPG was significantly improved with the leaf extract. Moreover, mixed extracts, such as SH82 and SH55, demonstrated enhanced activity proportional to the higher concentration of leaves. Remarkably, SH28 exhibited a substantial increase of 70% (*p* < 0.001), surpassing the activity of the leaf extract alone.

Conversely, the impact on bone-resorption-related gene expression, with the exception of RANK, TRAP, and TRAF6, was not markedly pronounced for either the F or L extract. Furthermore, mixed extracts, such as SH82 and SH55, displayed enhanced activity in proportion to higher concentration of leaves. Particularly, SH28 demonstrated a significant reduction (*p* < 0.001), surpassing the activity of the leaf extract alone.

## 4. Discussion

Osteoporosis, a prevalent condition in middle-aged and older women, remains a challenge in terms of a complete understanding of its pathophysiology, despite advancements in prevention and treatment strategies [20]. Achieving a delicate balance between bone formation and resorption is crucial in managing this metabolic bone disease.

Throughout the centuries, the therapeutic potential of bioactive compounds sourced from plants and natural origins has gained recognition for treating various ailments. These include tonic diseases, bone disorders, skin conditions, cancer, microbial infections, and metabolic or degenerative diseases [21]. Notably, there is growing interest in exploring the efficacy of natural substances with minimal or no side effects, especially for the prolonged administration required in preventing and treating conditions like osteoporosis [22].

Indeed, it is widely acknowledged that individuals with osteoporosis necessitate ongoing and lifelong treatment, often involving the use of two or more medications to mitigate the risk of fractures.

SH has gained considerable recognition in traditional oriental medicine for its therapeutic properties, particularly in addressing conditions like arthritis and neuralgia using various parts, including the roots, leaves, and fruits [23]. However, the specific impact of SH, especially when considering the individual parts, such as fruits and leaves, or their combined usage, on bone formation remains insufficiently explored. To fill this knowledge gap, we conducted this study to provide insights into this specific aspect.

In our investigation, we initially examined the ingredients present in SH F and L and their influence on osteogenic activity. Through a comparison of the components revealed in the HPLC chromatogram, we observed that peaks of phenolic acids, such as gallic acid and caffeic acid, were predominantly found in SH F, with minimal identification of flavonoid compounds. Conversely, the leaf extract exhibited peaks of a more diverse range of components, particularly in the area where flavonoids were detected.

Furthermore, both leaves and fruits of SH were found to contain chlorogenic acids (CGAs), with leaves exhibiting a sevenfold higher concentration than fruits. The TPCs were also approximately seven times higher in leaves. This heightened antioxidant activity in leaves was corroborated by results from DPPH and ROS generation inhibition tests. Moreover, leaf extracts rich in polyphenols showed higher TRAP inhibitory activity, stronger upregulation of osteoblast-related genes (Runx2, ALP, Coll-I), and more effective suppression of osteoclast-associated genes. This supports the view that the higher polyphenol content in SH L is functionally linked to their superior bone-regulating effects.

These findings underscore the distinct composition and antioxidant capabilities of SH L and F. Such variations in the polyphenols, flavonoids, gallic acid, and triterpenes content between leaves and fruits align with observations in most plant species. The bioactivity spectrum of phenolic compounds is influenced by their chemical structure, concentration, and interactions with other compounds. As SH L and F possess different matrices, their bioavailability and mechanisms of action are likely diverse [24]. Despite significant compositional differences, both the leaves and fruits demonstrated noteworthy osteogenesis-promoting activity. Interestingly, although the leaf activity appeared slightly higher than that of fruits, it did not seem to be directly correlated with the concentration of specific active components like CGA or rutin. Previous studies [15] have suggested that certain flavonoids in SH leaves may possess anti-inflammatory and osteogenic properties. Additionally, flavonoids such as quercetin, kaempferol, and rutin have been implicated in bone resorption signal pathway regulation, as well as bone formation [25]. However, this study revealed a remarkable activity in fruits despite low or barely detectable levels of flavonoid compounds, suggesting that the effect may arise from the independent or synergistic action of various ingredients rather than any specific compound.

In addition to assessing the impact of SH extract on bone formation, our investigation extended to its effect on bone resorption. In osteoporosis, the differentiation and maturation of osteoclasts play a pivotal role, as an imbalance with osteoclast production surpassing bone formation leads to a progressive loss of bone mass. Our findings revealed a significant reduction in the proliferation of osteoclasts in RAW264.7 cells treated with extracts from SH fruit or leaves. Interestingly, the tartrate-resistant acid phosphatase (TRAP) inhibitory activity of the leaf extract consistently surpassed that of the fruit extract. The outcomes concerning the expression of genes associated with osteoblastogenesis and osteoclastogenesis in leaf and fruit extracts of SH exhibited distinct characteristics. Primarily, both samples exerted a greater influence on the regulation of genes related to bone formation compared to bone resorption. Secondly, they upregulated the expression of genes predominantly involved in the early stages of differentiation. Thirdly, the leaf extract consistently demonstrated higher activity across most measured genes compared to the fruit extract. A noteworthy observation is the similar positive activity exhibited by extracts from leaves and fruits with different compositions against two enzymes, ALP and TRAP, which have entirely different functions. This phenomenon could potentially be explained as a manifestation of the multi-component, multi-target theory advocated in Oriental medicine.

In a subsequent investigation, we explored the osteogenic activity of mixed fruit and leaf extracts, comparing them with single extract treatments. MC3T3-E1 and RAW264.7 cells were subjected to 8:2 (SH82), 5:5 (SH55), and 2:8 (SH28) mixed extracts, as well as single extracts, to assess their impact on cell viability and morphological characteristics. Despite minimal effects on the growth of MC3T3-E1 and RAW264.7 cells, the osteogenic efficacy of the mixed extracts increased proportionally with the ratio of highly active leaf extract. Intriguingly, SH28 demonstrated a higher synergistic effect than leaves alone, suggesting potential mechanisms for increased enzyme activity, either through enhanced expression or activation by specific activators present in the additive. It is noteworthy that SH, when mixed with leaves and fruits in a cell culture system, demonstrated safety, reinforcing its potential application in further studies and clinical contexts. The osteogenic, or bone-formation-promoting, effect of the mixed extracts displayed a dependence on the ratio of the highly active leaf extract. Interestingly, SH28 exhibited an unexpectedly higher synergistic effect compared to leaves alone. In the context of the increased enzyme activity in vivo, two plausible explanations include heightened expression of the enzyme itself and activation by a specific activator present in the additive. In this study, the former hypothesis is considered plausible, as the gene expression of ALP and tartrate-resistant acid phosphatase (TRAP) enzymes exhibited a corresponding pattern of increase or decrease consistent with changes in their activity. Nevertheless, given the diverse components present in the extract, the potential contribution of the latter cannot be unequivocally ruled out.

Notably, the TRAP inhibitory activity of the mixed extract demonstrated a pattern opposite to the osteogenesis-promoting effect observed in ALP. In RAW264.7 cells, treatment with the mixed extract significantly reduced TRAP activity and the number of TRAP-positive multinucleated cells. Notably, the SH28 extract treatment group exhibited the highest inhibitory activity.

The impact of SH mixed extract on gene expression yielded distinct results compared to the individual leaf or fruit extracts. Specifically, while treatment with single extracts predominantly increased the expression of genes associated with the early stages of bone formation, such as Runx2, ALP, and Coll-I, the mixed extract upregulated the expression of all tested genes, including OPG and Bglab, irrespective of their designation as early or late markers of osteoblastogenesis (Figure 8). Unlike treatment with leaves or fruits alone, the mixed extract effectively downregulated the expression of all tested genes related to osteoclast proliferation and differentiation, such as TRAP, TRAF6, RANK, Ctsk, c-fos, and NFTAcl. These observations may align with the temporal expression patterns observed during induced cell differentiation in osteogenic medium.

While the data do not definitively determine which specific substances activated particular pathways, it is clear that SH extract was associated with the activation of bone formation-related pathways, and the combined extract of fruits and leaves further enhanced this activity. To identify the components of SH contributing to this effect, the major identified compounds were evaluated for their influence on ALP and TRAP enzyme activity and gene expression. The results showed that neo-chlorogenic acid (neo-CGA), chlorogenic acid (CGA), and rutin significantly increased ALP activity and its gene expression. In contrast, crypto-CGA and luteolin-7-O-glucoside exhibited relatively lower effects in the same assays. These findings indicate that among the tested compounds, neo-CGA, CGA, and rutin were more closely associated with promoting osteogenic markers under the experimental conditions.

## 5. Conclusions

Through this study, it was confirmed that, although SH leaves and fruits have completely different components and contents, both have the effect of promoting bone formation and that the osteogenic activity is much higher in the leaves. Furthermore, SH fruit-leaf mixed extract (SH28) showed a synergistic effect in promoting proliferation and mineralization of osteoblasts and inhibiting differentiation of osteoclasts.

Considering the important role of osteoblasts and osteoclasts in maintaining bone health, SH28, a mixed extract of SH fruits and leaves, could be an effective health supplement for the prevention and treatment of osteoporosis. However, this study was limited to in vitro experiments and has not yet been validated in animal models. Although key bioactive compounds were identified, the underlying molecular mechanisms remain unclear. Clinical trials are needed to confirm the efficacy and safety of SH28 in humans.

## Figures and Tables

**Table 1:** Content of several phytochemicals in SH F and L.

SH	CGA	Rut	L-7-G	TPC	5-HMF
	(μg/g)	(μg/g)	(μg/g)	(mg GAE/g)	(μg/g)
SH F	360 ± 12	T *	T	1.02 ± 0.04	112 ± 11
SH L	2395 ± 20	264 ± 65	86 ± 10	7.66 ± 0.07	ND

* T, trace amount.

**Table 2:** The preparation of SH Fand L mixtures.

**Samples**	**Mixing Ratio (%)**	**Solid Content (mg/g)**	**CGAs (μg/g)**
F	F 100	436	360
L	L 100	341	2395
SH82	F 80 + L 20	367	767
SH55	F 50 + L 50	451	1378
SH28	F 20 + L 80	428	1988

## Data Availability

The original contributions presented in this study are included in the article/Supplementary Materials. Further inquiries can be directed to the corresponding authors.

## Supplementary Materials

The following supporting information can be downloaded at https://www.mdpi.com/article/10.3390/biom15060844/s1, Table S1: Primer list; Figure S1: SH F (A) and L (B); Figure S2: Typical chromatograms of the extracts of SH F and L; Figure S3: Antioxidant activity of SH L and F; Figure S4: MTT assays for the MC3T3-E1 cell line; Figure S5: MTT assays for the Raw 264.7 macrophage cells. Figure S6: Typical chromatograms of the extracts of SH F, L and the mixed samples. Figure S7: MTT assay for the MC3T3-E1 cell line for SH F, L, and mixed samples. Figure S8: MTT assay for Raw 264.7 macrophage cells SH F, L, and mixed samples. References [26,27,28,29,30,31,32,33,34] are cited in the supplementary materials.
