# Supplementary material for: Synergistic Effect of Stauntonia hexaphylla (Thunb.) Decne Fruit and Leaf on RAW 264.7 Osteoclast and MC3T3-E1 Osteoblast Differentiation
Source: Biomolecules. 2025 Jun 10;15(6):844. doi: 10.3390/biom15060844 (PMC12190245; doi:10.3390/biom15060844)
Supplement: Supplementary file 1 [file biomolecules-15-00844-s001.zip › biomolecules-3591476-supplementary.pdf]

Supplementary Table S1. Primer list.

Gene	Forward primer	Reverse primer	Reference
ß-Actin	ATGAAGTGTGACGTTGACATCC	CCTAGAAGCATTTGCGGTGCACGATG	1
ALP	CGAGCAGGAACAGAAGTTTGC	TGGCCAAAAGGCAGTGAATAG	2
Runx2	ATGGCCGGGAATGATGAGAA	TCTGTCTGTGCCTTCTTGGT	
Col1-a1	GATGGATTCCAGTTCGAGTATG	GTTTGGGTTGCTTGTCTGTTTG	
OPG	ACAATGAACAAGTGGCTGTGCTG	CGGTTTCTGGGTCATAATGCAAG	3
RANKL	GCAGCATCGCTCTGTTCCTGTA	GCATGAGTCAGGTAGTGCTTCTGTG	4
BGLAP	GTGCAGACCTAGCAGACACCA	GTAGCGCCGGAGTCTATTCA	5
TRAP	CTGCTGGGCCTACAAATCAT	GGTAGTAAGGGCTGGGGAAG	6
RANK	AGAAGACGGTGCTGGAGTCT	TAGGAGCAGTGAACCAGTCG	7
MMP9	GACGGCACGCCTTGGTGTAG	AGGAGCGGCCCTCAAAGATG	
Ctsk	ATATGTGGGCCACCATGAAAGTT	TCGTTCCCCACAGGAATCTCT	
c-fos	GGGACAGCCTTTCCTACTACC	GATCTGCGCAAAAGTCCTGT	8
NFATc1	GGTAACTCTGTCTTTCTAACCTTAAGCTC	GTGATGACCCCAGCATGCACCAGTCACAG	9

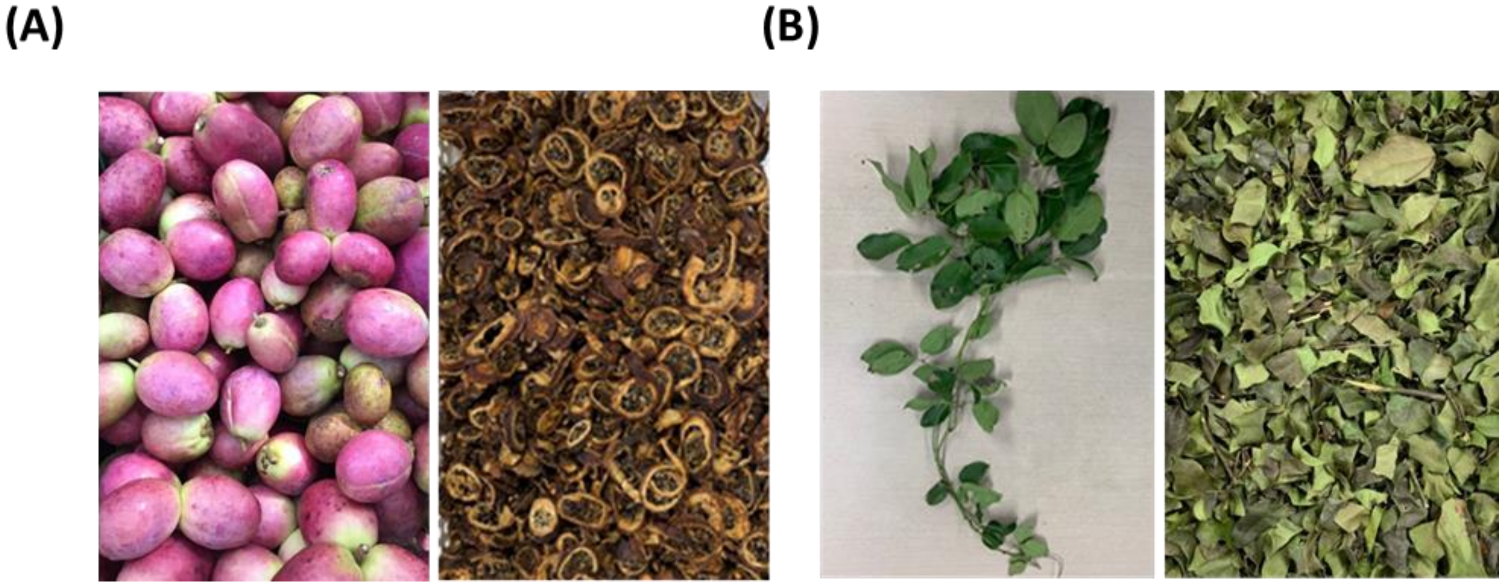

Supplementary Figure S1. SH fruits (A) and leaves (B).

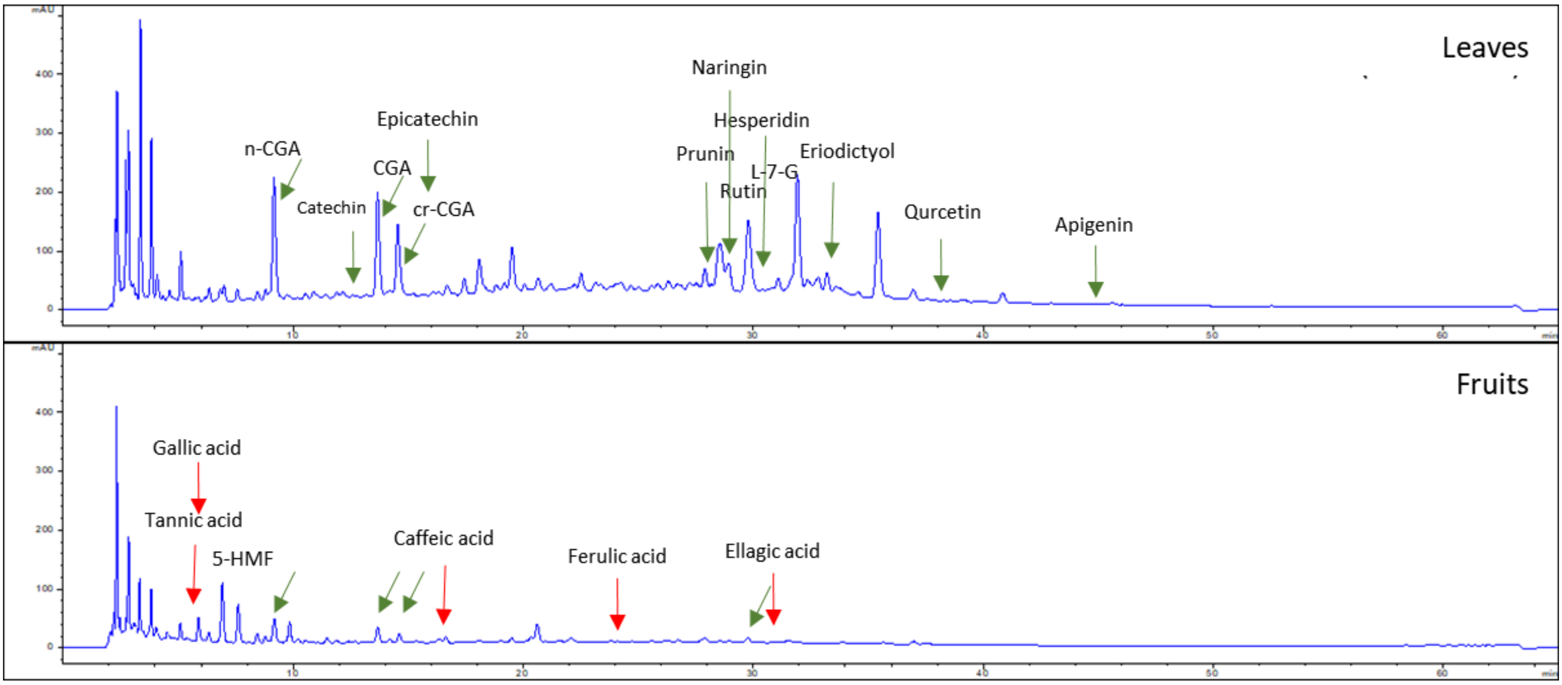

Supplementary Figure S2. Chromatograms of the extracts of SH fruits and leaves.

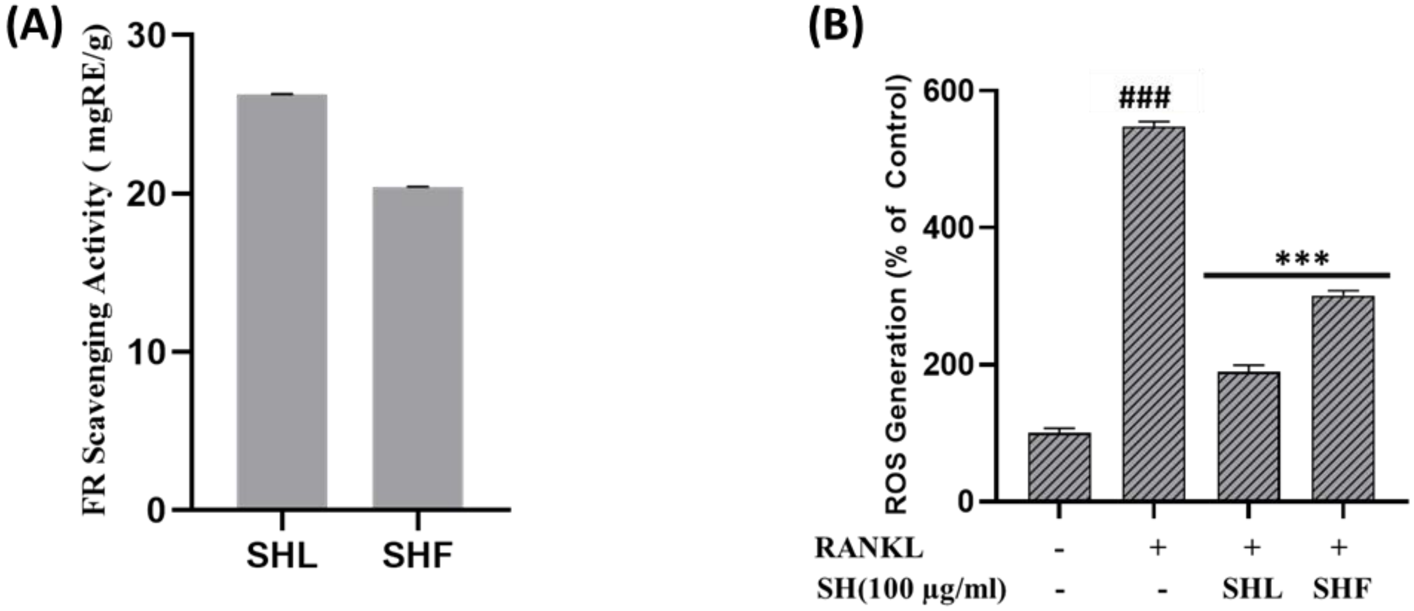

Supplementary Figure S3. Antioxidant activity of SH leaf and fruit as evaluated by (A) the DPPH assay and (B) Cellular ROS detection via DCF-DA reagent in RAW 264.7 cells. ###p<0.001 compared to control, ***p<0.001 compared to RANL treatment.

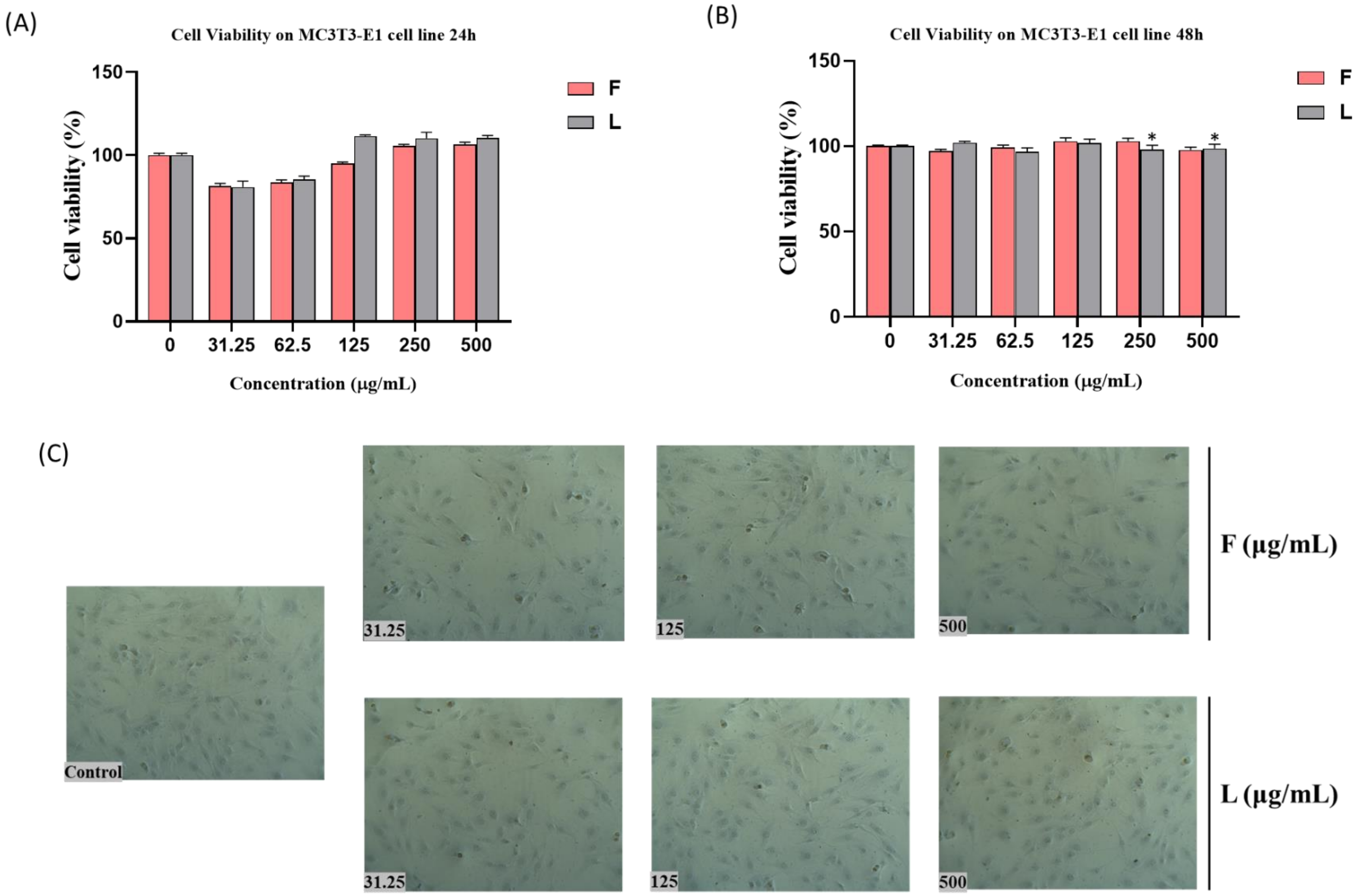

Supplementary Figure S4. MTT assays for the MC3T3-E1 cell line (A) Assessment of cell viability in MC3T3-E1 pre-osteoblast cells (1×10[4] cells/well) treated with varying concentrations of SHL and SHF (0–500 µg/ml) for 24h (B) Assessment of cell viability in MC3T3-E1 pre-osteoblast cells (1×10[4] cells/well) treated with varying concentrations of SHL and SHF (0–500 µg/ml) for 48h. (C) Visualization of cellular morphology through phase-contrast microscopy (×20 magnification) after the 2-day treatment with SHF. Scale bar = 100 µm. The presented data represents the mean ± standard deviation (SD) from three independent experiments. Statistical analysis indicated significant differences, marked as *$p<0.05$, **$p<0.01$, ***$p<0.001$ in comparison with the reference groups treated with ascorbic acid and β-glycerophosphate, as indicated.

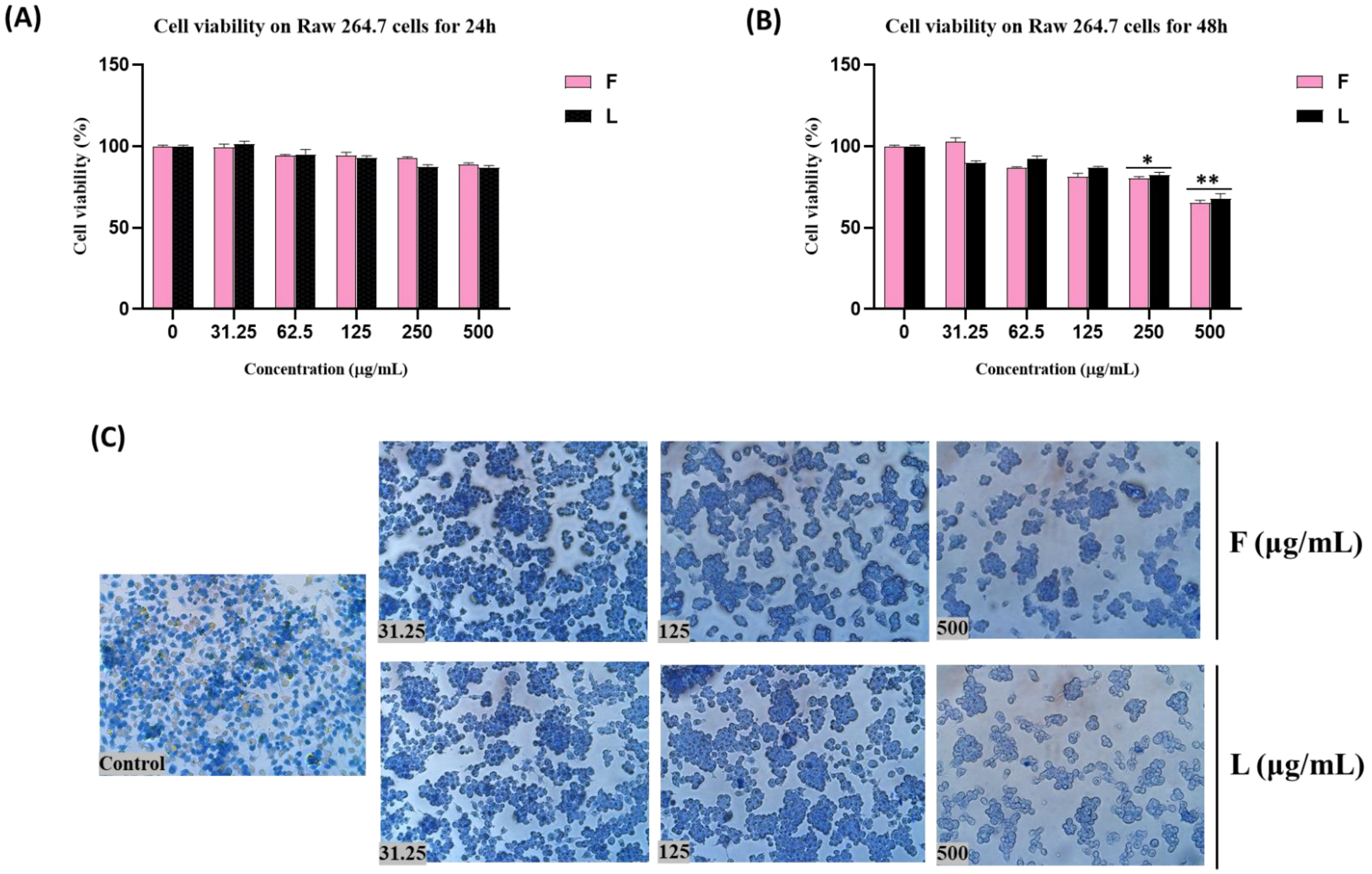

Supplementary Figure S5. Raw 264.7 macrophage cells (1×10^4 cells/ml) were exposed to varying concentrations of SHF (0–500 μg/ml) for a duration of 2 days. (A) Cell viability was assessed through an MTT assay at 24h and (B) 48h. (C) Cellular images were captured using phase-contrast microscopy (× 20 magnification). Scale bar = 100 μm. The presented data represents the mean ± standard deviation (SD) from three independent experiments. Statistical analysis disclosed significant differences, denoted as *$p<0.05$, **$p<0.01$ compared to the control.

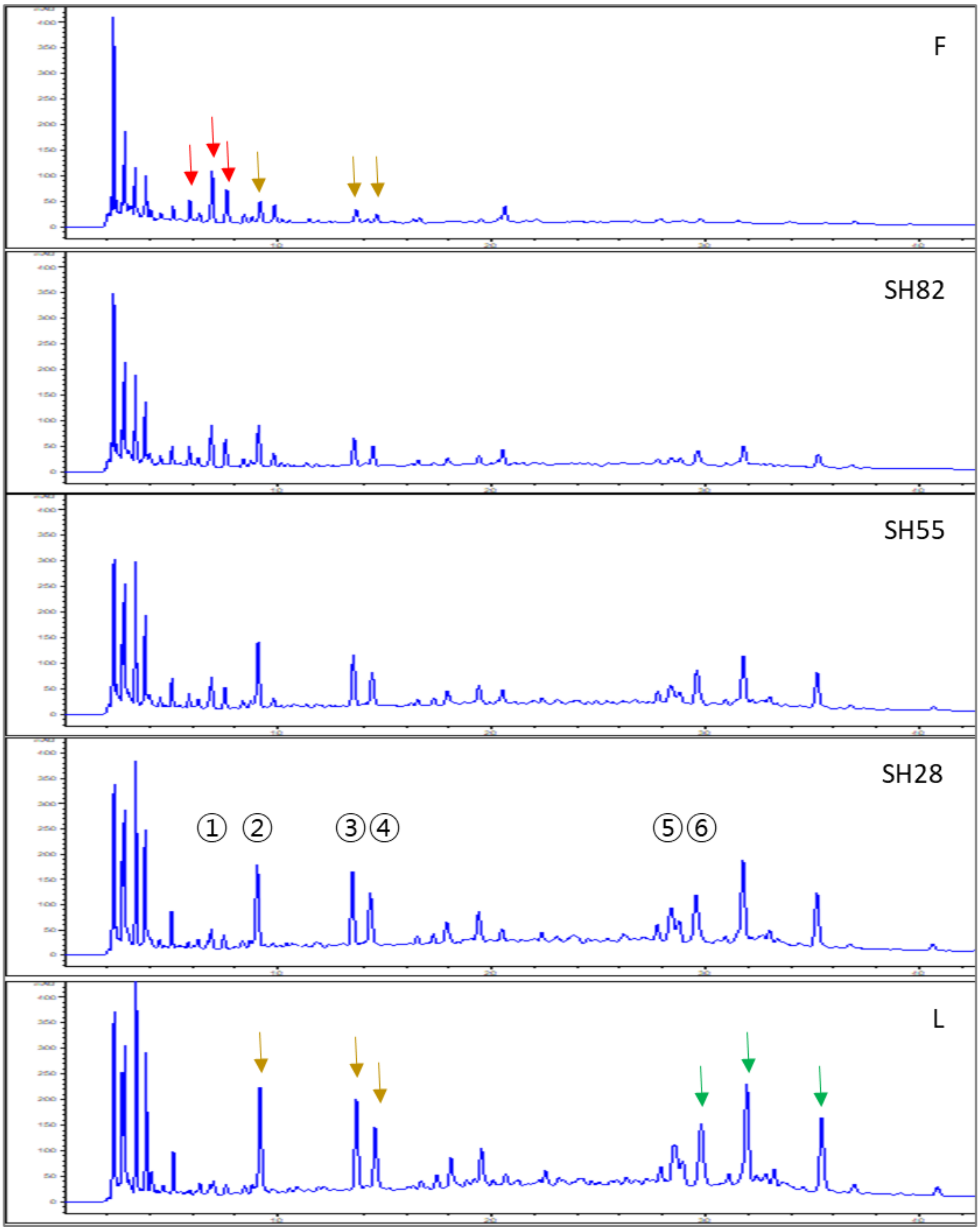

Supplementary Figure S6. Typical chromatograms of the extracts of fruits, leaves, and the mixed samples.

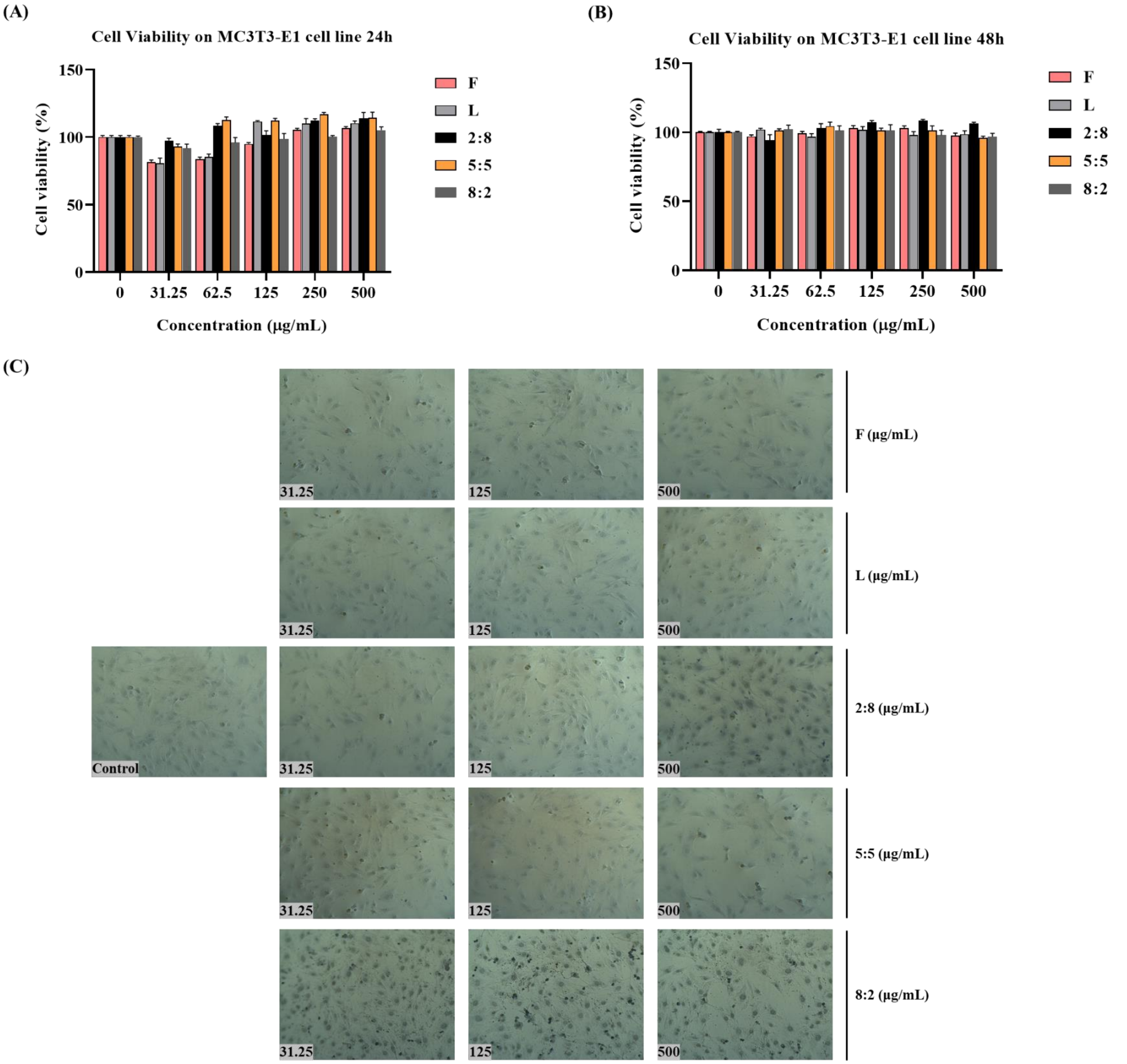

Supplementary Figure S7. MTT assays for the MC3T3-E1 cell line (A) Assessment of cell viability in MC3T3-E1 pre-osteoblast cells (1×10[4] cells/well) treated with varying concentrations of SHL and SHF (0–500 μg/ml) for 24h (B) Assessment of cell viability in MC3T3-E1 pre-osteoblast cells (1×10[4] cells/well) treated with varying concentrations of SHL and SHF (0–500 μg/ml) for 48h. (C) Visualization of cellular morphology through phase-contrast microscopy (×20 magnification) after the 2-day treatment with SHF. Scale bar = 100 μm. The presented data represents the mean ± standard deviation (SD) from three independent experiments. Statistical analysis indicated

significant differences, marked as *p<0.05, **p<0.01, ***p<0.001 in comparison with the reference groups treated with ascorbic acid and β-glycerophosphate, as indicated.

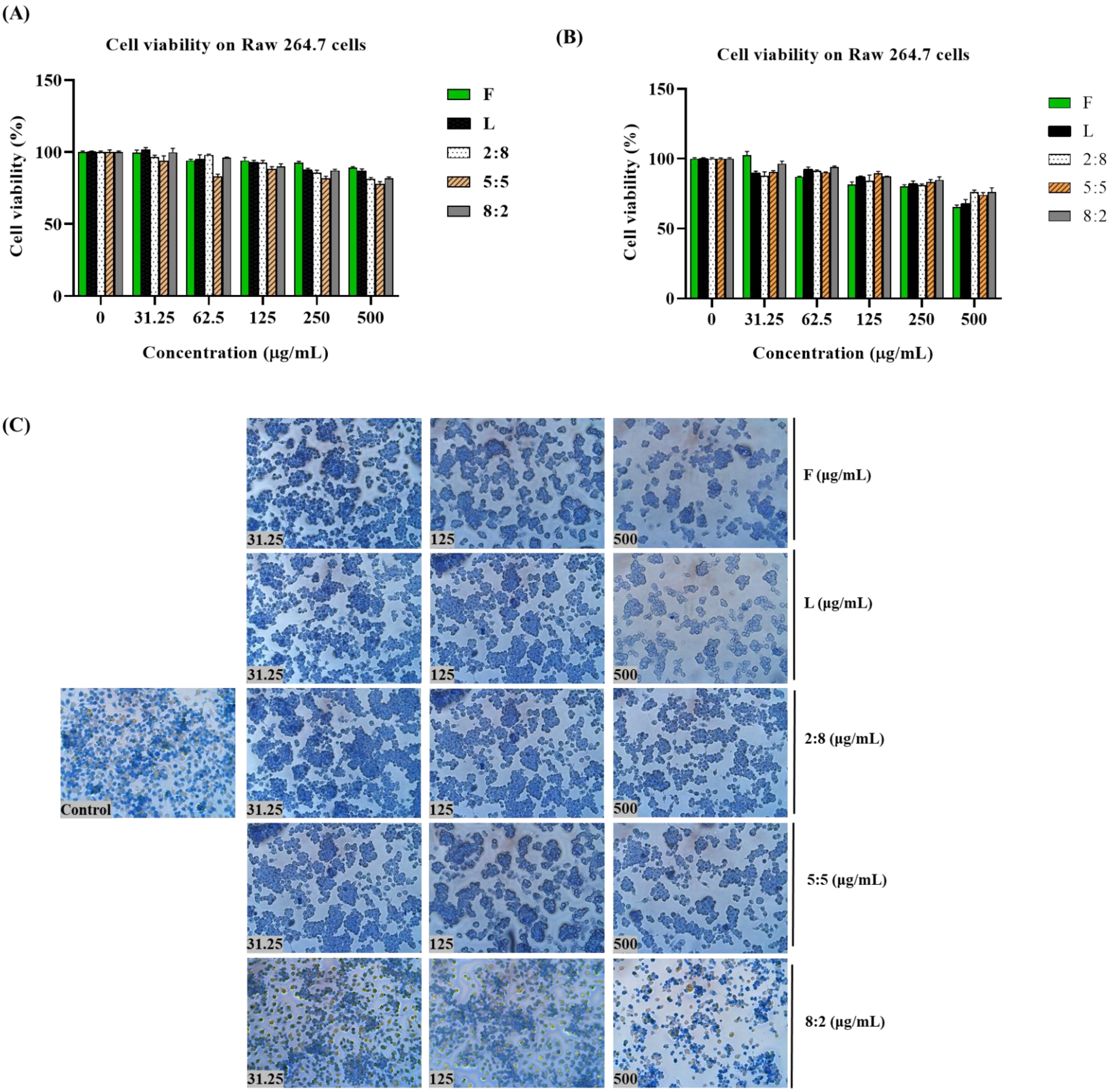

Supplementary Figure S8. Raw 264.7 macrophage cells (1×10^4 cells/ml) were exposed to varying concentrations of SHF (0–500 μg/ml) for a duration of 2 days. (A) Cell viability was assessed through an MTT assay at 24h and (B) 48h. (C) Cellular images were captured using phase-contrast microscopy (× 20 magnification). Scale bar = 100 μm. The presented data represents the mean ± standard deviation (SD) from three independent experiments. Statistical analysis disclosed significant differences, denoted as *p<0.05, **p<0.01 compared to the control.

References

(1) Siraj, F. M.; SathishKumar, N.; Kim, Y. J.; Kim, S. Y.; Yang, D. C. Ginsenoside F2 possesses anti-obesity activity via binding with PPARγ and inhibiting adipocyte differentiation in the 3T3-L1 cell line. *Journal of enzyme inhibition and medicinal chemistry* **2015**, *30* (1), 9-14.

(2) Siddiqi, M. H.; Siddiqi, M. Z.; Ahn, S.; Kim, Y.-J.; Yang, D. C. Ginsenoside Rh1 induces mouse osteoblast growth and differentiation through the bone morphogenetic protein 2/runt-related gene 2 signalling pathway. *Journal of Pharmacy and Pharmacology* **2014**, *66* (12), 1763-1773.

(3) Zhao, J.-J.; Wu, Z.-F.; Wang, L.; Feng, D.-H.; Cheng, L. MicroRNA-145 mediates steroid-induced necrosis of the femoral head by targeting the OPG/RANK/RANKL signaling pathway. *PLoS One* **2016**, *11* (7), e0159805.

(4) Zhou, L.-P.; Wong, K.-Y.; Yeung, H.-T.; Dong, X.-L.; Xiao, H.-H.; Gong, A. G.-W.; Tsim, K. W.-K.; Wong, M.-S. Bone protective effects of danggui buxue tang alone and in combination with tamoxifen or raloxifene in vivo and in vitro. *Frontiers in pharmacology* **2018**, *9*, 779.

(5) Skafi, N.; Abdallah, D.; Soulage, C.; Reibel, S.; Vitale, N.; Hamade, E.; Faour, W.; Magne, D.; Badran, B.; Hussein, N. Phospholipase D: A new mediator during high phosphate-induced vascular calcification associated with chronic kidney disease. *Journal of cellular physiology* **2019**, *234* (4), 4825-4839.

(6) Li, L.; Yang, M.; Shrestha, S. K.; Kim, H.; Gerwick, W. H.; Soh, Y. Kalkitoxin reduces osteoclast formation and resorption and protects against inflammatory bone loss. *International Journal of Molecular Sciences* **2021**, *22* (5), 2303.

(7) Lee, Y. D.; Yoon, S.-H.; Ji, E.; Kim, H.-H. Caveolin-1 regulates osteoclast differentiation by suppressing cFms degradation. *Experimental & Molecular Medicine* **2015**, *47* (10), e192-e192.

(8) Bougault, C.; Aubert-Foucher, E.; Paumier, A.; Perrier-Groult, E.; Huot, L.; Hot, D.; Duterque-Coquillaud, M.; Mallein-Gerin, F. Dynamic compression of chondrocyte-agarose constructs reveals new candidate mechanosensitive genes. *PloS one* **2012**, *7* (5), e36964.

(9) Hajavifard, N.; Matinhomaee, H.; Hosseini, S. A. Effect of Aerobic Training and Vitamin D Consumption on NFATc1 Gene Expression in Bone Tissue of Rats Exposed to H2O2. *Gene, Cell and Tissue* **2020**, *7* (4).
